# Osteosarcoma cells depend on MCL-1 for survival, and osteosarcoma metastases respond to MCL-1 antagonism plus regorafenib in vivo

**DOI:** 10.1186/s12885-024-13088-7

**Published:** 2024-11-04

**Authors:** Yanhao Ji, Michael A. Harris, Lucas M. Newton, Tiffany J. Harris, W. Douglas Fairlie, Erinna F. Lee, Christine J. Hawkins

**Affiliations:** 1 Department of Biochemistry and Chemistry, La Trobe Institute for Molecular Science, La Trobe University, Melbourne, VIC 3086 Australia; 2 Peter MacCallum Cancer Centre, Melbourne, VIC 3000 Australia; 3 Swinburne University, Hawthorn, VIC 3122 Australia; 4 Olivia Newton-John Cancer Research Institute, Heidelberg, VIC 3084 Australia; 5 School of Cancer Medicine, La Trobe University, Melbourne, VIC 3086 Australia

**Keywords:** Osteosarcoma, Sarcoma, BH3-mimetic, MCL-1, BCL-x_L_, Kinase inhibitor, S63845, Regorafenib

## Abstract

Osteosarcoma is the most common form of primary bone cancer, which primarily afflicts children and adolescents. Chemotherapy, consisting of doxorubicin, cisplatin and methotrexate (MAP) increased the 5-year osteosarcoma survival rate from 20% to approximately 60% by the 1980s. However, osteosarcoma survival rates have remained stagnant for several decades. Patients whose disease fails to respond to MAP receive second-line treatments such as etoposide and, in more recent years, the kinase inhibitor regorafenib. BCL-2 and its close relatives enforce cellular survival and have been implicated in the development and progression of various cancer types. BH3-mimetics antagonize pro-survival members of the BCL-2 family to directly stimulate apoptosis. These drugs have been proven to be efficacious in other cancer types, but their use in osteosarcoma has been relatively unexplored to date. We investigated the potential efficacy of BH3-mimetics against osteosarcoma cells in vitro and examined their cooperation with regorafenib in vivo. We demonstrated that osteosarcoma cell lines could be killed through inhibition of MCL-1 combined with BCL-2 or BCL-x_L_ antagonism. Inhibition of MCL-1 also sensitized osteosarcoma cells to killing by second-line osteosarcoma treatments, particularly regorafenib. Importantly, we found that inhibition of MCL-1 with the BH3-mimetic S63845 combined with regorafenib significantly prolonged the survival of mice bearing pulmonary osteosarcoma metastases. Together, our results highlight the importance of MCL-1 in osteosarcoma cell survival and present a potential therapeutic avenue that may improve metastatic osteosarcoma patient outcomes.

**Supplementary Information:**

The online version contains supplementary material available at 10.1186/s12885-024-13088-7.

## Background

Osteosarcoma is the most prevalent primary tumor of the bone and has its highest incidence rate in children and adolescents [1, 2]. The current standard-of-care for osteosarcoma patients is neoadjuvant and adjuvant chemotherapy coupled with surgery [3]. Chemotherapy for osteosarcoma patients is typically a combination treatment consisting of doxorubicin, cisplatin and high dose methotrexate, referred to as MAP [4]. The addition of MAP to surgery as a treatment for osteosarcoma patients saw the 5-year survival rate increase from approximately 20% to around 60% by the 1980s [5]. MAP (occasionally combined with ifosfamide) remains the first-line treatment for osteosarcoma [6]. However, there has been no significant increase in the survival rate for osteosarcoma patients in decades, and the 5-year survival rate for patients with metastatic osteosarcoma has remained stubbornly low, hovering around 30% [5]. For patients who do not respond to MAP, regorafenib is the only second-line osteosarcoma therapy that has demonstrated significant benefit in randomized clinical trials [7, 8]. The progression-free survival benefits conferred to bone tumor patients by regorafenib were recently recapitulated in “real world” contexts in multiple Canadian hospitals [9]. Regorafenib is an oral multi-kinase inhibitor, targeting both the tumor cells directly and the tumor microenvironment [10, 11]. By inhibiting key kinases involved in angiogenesis, oncogenesis and tumor immunity, such as vascular endothelial growth factor receptors (VEGFRs), tyrosine kinase with immunoglobulin-like and epidermal growth factor-like domains 2 (TIE2), KIT, RET, RAF1, BRAF, platelet-derived growth factor receptors (PDGFRs), fibroblast growth factor receptors (FGFRs) and colony-stimulating factor-1 receptor (CSF-1R) [10, 12], regorafenib exerts a broad-spectrum anti-cancer effect. This makes it an effective option for both mono- and combination therapy in various types of cancers. Apart from osteosarcoma, regorafenib is also used to treat metastatic colorectal cancer [13], gastrointestinal stromal tumors [14], hepatocarcinoma cancer [15], sarcoma [16] and other malignancies where these pathways are critically involved [11]. Although regorafenib extended the progression-free survival of metastatic osteosarcoma patients, it did not confer long term survival [7, 8]. Therefore, there is an urgent need for more effective treatment options, particularly for patients whose tumors do not respond to MAP.

One of the hallmarks of cancer is impaired cell death signaling, which can arise from an imbalance in pro- and anti-apoptotic protein expression or activity [17]. BCL-2 family proteins are key regulators of intrinsic apoptosis, and different sub-groups of these proteins are responsible for promoting or inhibiting cell death [18] (Supplementary Fig. 1). BCL-2 family proteins that inhibit cell death include MCL-1, BCL-x_L_ and BCL-2, and their expression has been implicated in poor cancer patient outcome and resistance to treatment [18]. The importance of BCL-2 family proteins in cancer survival spurred the development of a new class of therapeutics called “BH3-mimetics” that can directly inhibit their pro-survival activity, altering the balance of pro- and anti-apoptotic signaling to induce cell death [19] (Supplementary Fig. 1).

ABT-737, the first BH3-mimetic to be developed, induced tumor regression through inhibition of BCL-2, BCL-x_L_ and BCL-w [20]. As ABT-737 is not orally available, the bioavailable analog ABT-263 (navitoclax) was developed, which has a similar BCL-2 protein binding profile to ABT-737 [21]. Navitoclax displayed strong efficacy against a range of cancers with high levels of BCL-2 and BCL-x_L_ expression, most notably chronic lymphocytic leukemia (CLL), and lymphomas [22]. However, the strong affinity of navitoclax for BCL-x_L_ resulted in patients developing thrombocytopenia at high doses, due to the dependency of platelets on BCL-x_L_ for survival [23]. To circumvent thrombocytopenia, the BCL-2-specific BH3-mimetic ABT-199 (venetoclax) was developed [24]. Venetoclax is now used to treat patients with CLL [25] and acute myeloid leukemia (AML) [26]. Additional selective BH3-mimetics were also developed, such as A-1331852 [27] and S63845 [28], which inhibit BCL-x_L_ and MCL-1 respectively. The specificity of these agents has been since verified [29]. An orally available derivative of S63845 (MIK665) is under evaluation in clinical trials for the treatment of AML (NCT02979366 and NCT03672695), multiple myeloma and lymphoma (NCT02992483).

Few studies have explored whether BH3-mimetics present a viable treatment option for osteosarcoma. A small number of established osteosarcoma cell lines were moderately sensitive to killing with ABT-737 alone, but that drug exhibited synergy when used in combination with cisplatin [30]. WEHI-539, an inhibitor of BCL-x_L_, similarly manifested poor activity against osteosarcoma cells in vitro but sensitized cells to doxorubicin [31]. Another report showed combined inhibition of BCL-x_L_ and MCL-1 was effective at inducing cell death in two osteosarcoma cell lines, whereas both drugs were relatively ineffective on their own [32].

In this study, we initially compared the sensitivity of osteosarcoma cell lines to a panel of BH3-mimetics alone, and in combination with each other. We identified S68345 as a promising candidate to induce osteosarcoma cell death through inhibition of MCL-1. Combined treatment of osteosarcoma cells with S63845 and second-line osteosarcoma therapies resulted in cooperative killing of osteosarcoma cells, particularly with the multi-tyrosine kinase inhibitor regorafenib. Finally, we confirmed that the cooperation observed between the MCL-1 inhibitor S63845 and regorafenib in vitro could be replicated in vivo using a mouse model of metastatic osteosarcoma.

## Methods

### Cell culture and drugs

The drugs used in this study were S63845 (Catalog #S8383, Selleck Chemicals, Houston, TX, USA), A-1331852 (Catalog #S7801, Selleck Chemicals), ABT-199 (MedChem Express, Monmouth Junction, NJ, USA), etoposide (Catalog #S1225, Selleck Chemicals; Jomar Life Research), regorafenib (Catalog #S1178, Selleck Chemicals), and Q-VD-OPh (Catalog #S7311, Selleck Chemicals). For in vivo studies, S63845 was prepared in 25 mmol/L HCl, 20% 2-hydroxy propyl-β-cyclo dextrin; regorafenib was prepared in 42.5% 1,2 Propylenglycol (1,2-Propandiol), 42.5% PEG 400, 15% Pluronic F68, 20% ddH_2_O.

Human osteosarcoma cell lines KHOS, KRIB and 143B were provided by Nicholas Saunders (University of Queensland). SJSA-1 and U2OS cells were provided by Damian Myers (University of Melbourne). MG-63 cells were provided by Elena Ivanova (RMIT University). All cell lines were grown in DMEM (Catalog #11995073, Thermo Fisher Scientific; Waltham, MA, USA) containing 10% FCS at 37^o^C and 5% CO_2_ in a humidified chamber. All cell lines were confirmed to be negative for mycoplasma and were authenticated by short tandem repeat profiling.

### CellTiter-Glo assays

Two thousand cells were seeded into white 96-well plates. Cell viability was measured with CellTiter-Glo 2.0 (Catalog #G9243, Promega, Madison, WI, USA) according to the manufacturer’s instructions. Luminescence was measured on SpectraMax M5e plate reader (Molecular Devices, San Jose, CA, USA).

### Flow cytometry

Annexin V-FITC/Propidium Iodide staining was measured as previously described [33]. Briefly, 25,000 cells were seeded per well into 96-well plates and left to adhere overnight. Media was replaced with media alone or media containing drugs and left for 2–6 h. For analysis of apoptosis, cells were harvested and resuspended in 50 µg/ml propidium iodide (Catalog #P4864, Sigma-Aldrich, St. Louis MO, USA) and 750 ng/mL Annexin V-FITC (Catalog #556419, BD Biosciences; Franklin Lakes, NJ, USA) diluted in binding buffer (10 mM HEPES, 140 mM NaCl, 2.5 mM CaCl_2_, pH 7.4) and incubated for 10 min. Stained samples were analysed using a FACS Canto™ flow cytometer (BD Biosciences). The percentages of Annexin V-FITC-positive, PI-positive, and double-positive cells were calculated using FlowJo 10 software (BD Biosciences).

### SDS-PAGE and immunoblotting

Cells were lysed in radioimmunoprecipitation assay (RIPA) buffer (150 mM sodium chloride, 1.0% Triton X-100, 0.5% sodium deoxycholate, 0.1% SDS, 50 mM Tris, pH 8.0) supplemented with protease inhibitor cocktail (Catalog #P8340, Sigma-Aldrich). Protein concentrations in cell lysates were determined using a Micro BCA assay kit (Catalog #23235, Thermo Fisher Scientific). For each sample, 20 µg of total protein was loaded. Proteins were separated using 12% SDS-PAGE gels and transferred to Hybond PVDF 0.22 μm membranes (Catalog #GE10600021, GE Healthcare Life Science, Marlborough, MA, USA). Membranes were blocked with EveryBlot blocking buffer (Catalog #12010020, Bio-Rad, Hercules, CA, USA) for 10 min at room temperature and probed with the following primary antibodies diluted in EveryBlot blocking buffer: BCL-2 (Catalog #ab182858, Abcam, Cambridge, UK; 1:2000), BCL-x_L_ (Catalog # 551020, BD BioSciences, Macquarie Park, NSW, Australia; 1:1000), MCL-1 (Catalog #ab243136, Abcam; 1:1000), Bmf (Catalog #ALX-804-343, Enzo Life Sciences, Farmingdale, NY, USA; 1:1000) BIM (Catalog #2819, Cell Signaling Technology, Danvers, MA, USA; 1:1000), BIK (Catalog #4592, Cell Signaling Technology; 1:1000), BID (Catalog #AF860, R&D Systems, Minneapolis, MN, USA; 1:400), PUMA (Catalog #ab9643, Abcam; 1:1000), BAD (Catalog #32445, Abcam; 1:1000), BAK (Catalog # AM03, Merck Millipore Burlington, MA, USA; 1:50), BAX (Catalog #ABC11, Merck Millipore; 1:1000), ACTIN (Catalog #A5316, Sigma-Aldrich; 1:3000) and secondary antibodies anti-rabbit HRP (Catalog #7074, Cell Signaling Technology; 1:10,000), anti-mouse HRP (Catalog # A9044, Sigma-Aldrich; 1:10,000), anti-rat HRP (Catalog #GENA935, GE Healthcare Life Science; 1: 10,000), anti-goat HRP (Catalog #31400, Thermo Fisher Scientific; 1: 10,000).

### Animal studies

Animal experiments were conducted in accordance with the Australian Code of Practice for the Care and Use of Animals for Scientific Purposes, as approved by the La Trobe Animal Ethics Committee (project #AEC22036). Five- to six-week-old BALB/c-Foxn1^nu/ARC^ (nude) mice were purchased from the Animal Resource Centre/Ozgene and housed at La Trobe University Animal Research and Teaching Facility. Mice were kept in individual ventilated cages with unrestricted access to food and water and monitored daily. Natural Killer cells were transiently depleted by intraperitoneal injection of 50 µl of anti-asialo-GM1 antibody (Catalog #986-10001, Wako, Osaka, Japan) 24-hours prior to tumor implantation, as previously described [34]. Pulmonary tumor models were established by injecting 50,000 luciferase-expressing 143B cells [33] suspended in 100 µl of PBS into the tail vein using a 26-guage needle. One week after tumor injection, mice were imaged twice a week using an IVIS Lumina XR III (Perkin Elmer, Waltham, MA, USA) to monitor lung tumor growth. Briefly, mice were administered an intraperitoneal injection of 150 mg/kg of D-Luciferin, potassium salt (Catalog #122796, Perkin Elmer), and anesthetized using isoflurane; the luminescence intensity was measured every minute over 10 min with automatic exposure times. The highest luminescence within a defined circular region corresponding to lung tumors was recorded. When the highest luminescence of the lung region was first reached 1 × 10^5^ photons per second, the mice were alternatively allocated to each treatment group. We treated mice with regorafenib on days one, three and five of the week, and S63845 on days two and four, for three consecutive weeks. S63845 was administered at 25 mg/kg by intraperitoneal injection twice per week for three weeks. Regorafenib was administered at 30 mg/kg by oral gavage three times per week for three weeks. Mice were monitored for signs of compromised health every day, or twice a day if concerned, for up to twelve weeks. Mice were euthanized by CO_2_ asphyxiation when health conditions were assessed to meet criteria approved by the animal ethics committee or at the endpoint of the experiment.

### Statistics

GraphPad Prism 9.5.1 was used to perform the statistical and correlation analyses specified in the figure legends. Adjustments for multiple comparisons were achieved using either Sidak or Bonferroni corrections, as outlined in the legends.

## Results

### Osteosarcoma cells depend on MCL-1 for their survival

**Fig. 1:**
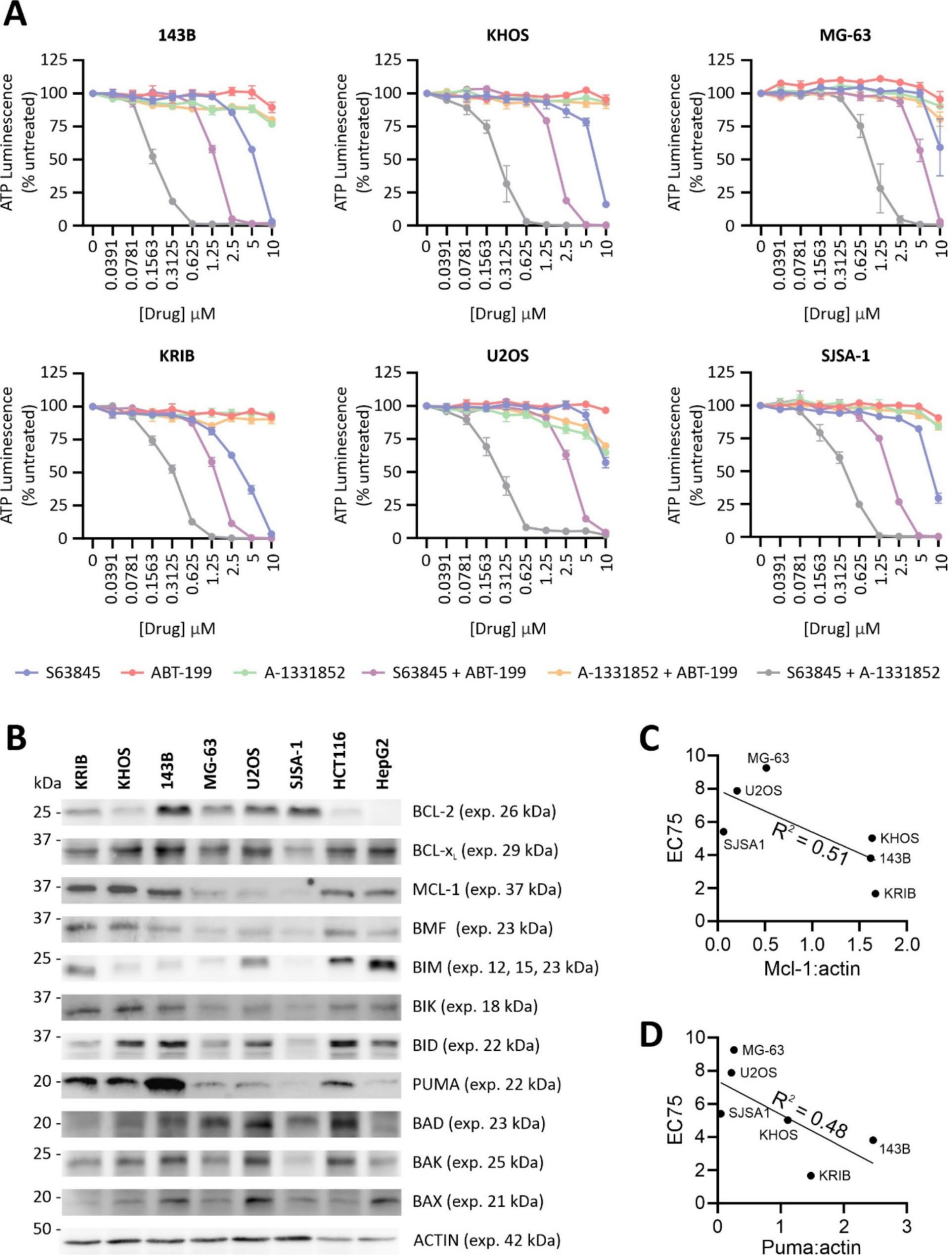
Inhibition of MCL-1 combined with BCL-x_L_ or BCL-2 reduces the viability of established osteosarcoma cells, and sensitivity to MCL-1 antagonism correlates with MCL-1 and PUMA expression. (**A**) The viability of six human osteosarcoma cell lines were assessed after 24-hour treatment with single and combined treatment with BH3 mimetic(s) using CellTiter-Glo, relative to DMSO in media controls. Data is presented as the mean +/- SEM of three independent replicates. (**B**) The expression levels of BCL-2 family proteins were assessed by immunoblotting. HCT116 and HepG2 cell lines were included as positive controls for the antibodies. Positions of molecular weight markers are shown to the left of the blots. The expected size of each protein is stated on the right. Full-length blots/gels are presented in Supplementary Figs. 2 and 3. (**C**, **D**) The ratios of sub-saturating signals from MCL-1 and ACTIN blots (**C**) or PUMA and ACTIN (**D**) blots were calculated and graphed against the concentrations at which the CellTiter-Glo luminescence in S63845-treated samples was 75% of untreated samples (EC75). GraphPad Prism was used to calculate R^2^ correlation coefficients

To investigate whether antagonizing BCL-2 family proteins can kill osteosarcoma cells, we tested the sensitivity of six established osteosarcoma cell lines to inhibition of BCL-2 (ABT-199), MCL-1 (S63845) and/or BCL-x_L_ (A-1331852) (Supplementary Fig. 1) for 24 h using CellTiter-Glo 2.0 cell viability assays. None of the cell lines responded to ABT-199 treatment (Fig. 1). High doses of A-1331852 slightly reduced the viability of U2OS cells, but this drug did not affect the other cell lines. However, low micromolar concentrations of S63845 reduced the viability of all cell lines, with 143B and KRIB cells being particularly sensitive. These findings suggest that MCL-1 plays a more prominent role in the survival of osteosarcoma cells than BCL-2 or BCL-x_L_. Combined treatment using high concentrations of A-1331852 and ABT-199 had minimal impact. In contrast, both A-1331852 and ABT-199 cooperated with S63845 to kill osteosarcoma cells more potently than S63845 alone, with the co-targeting of BCL-x_L_ providing the more dramatic effect. These results underscore the essential role of MCL-1 in the survival of osteosarcoma tumor cells and highlight the therapeutic potential of MCL-1 inhibition for osteosarcoma patients.

Published work suggests that the sensitivity to BH3-mimetics can depend on expression levels of the BCL-2 family proteins in some cancers. For example, prior data revealed that sensitivity to antagonists of BCL-2 or BCL-x_L_ most often correlated with expression levels of each target protein [35–38]. However, the osteosarcoma cells tested in this study failed to respond to ABT-199, regardless of BCL-2 expression (Fig. 1). U2OS cells were weakly sensitive to BCL-x_L_ inhibition, yet they expressed similar levels of BCL-x_L_ as the resistant lines. In previous studies focusing on other cancer types, responsiveness to MCL-1 inhibitors tended to mirror MCL-1 levels [39] and inversely correlate with BCL-x_L_ abundance [35, 40–44]. Consistent with that pattern, the three osteosarcoma cell lines with the highest MCL-1 expression were the most responsive to S63845, although BCL-x_L_ expression in this panel of cell lines failed to reflect S63845 sensitivity (Fig. 1B, C). MCL-1 expression has also been associated with resistance to agents targeting BCL-2 and/or BCL-x_L_ [45–47], however the osteosarcoma cells survived dual ABT-199/A-1331852 exposure regardless of their MCL-1 expression levels (Fig. 1). Sensitivity to BH3 mimetics could theoretically be boosted by high levels of pro-apoptotic BCL-2 relatives, which could potentially disable pro-survival relatives not targeted by the drug(s). Consistent with this notion, the cell lines that were more sensitive to S63845 (KRIB, KHOS and 143B) expressed higher levels of some BH3-only proteins (especially PUMA) than the more resistant cell lines (Fig. 1B, D).

**Fig. 2:**
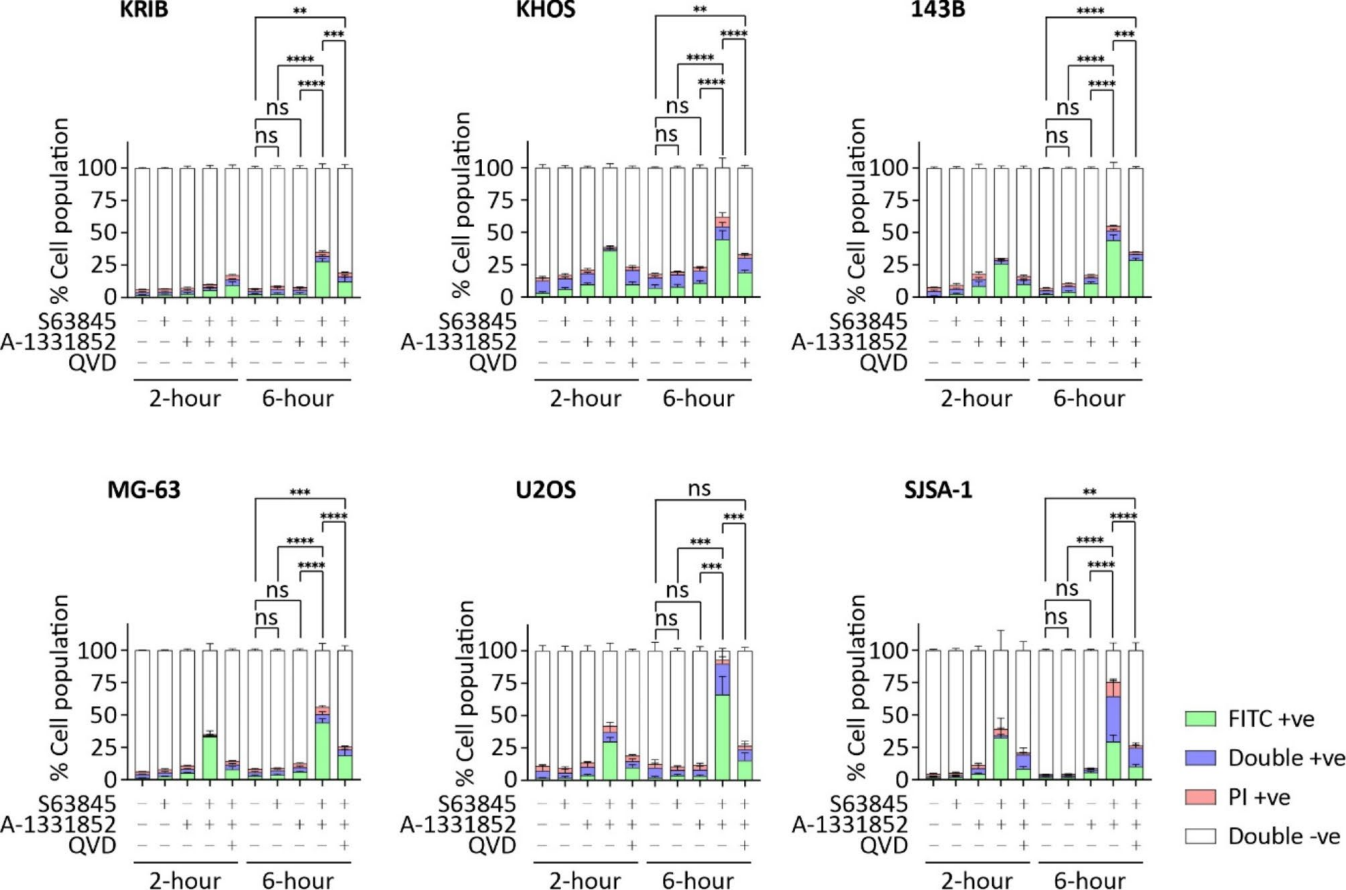
S63845 combined with A-1331852 induces cell death in vitro via a caspase-dependent manner. Specified osteosarcoma cells were cultured in growth media containing 1 µM of either S63845, A-1331852, or both, for either 2- or 6-hours. Pre-incubation with 10 µM Q-VD was conducted from 2-hours before treatment to the end of the entire treatment period. Cell death was measured with Annexin V/PI staining and quantitated by flow cytometry. Data represent mean ± SEM of three independent assays. Statistical analysis of differences in total positive events (combined FITC + ve, PI + ve and Double + ve) after 6-hours of treatment were compared using ANOVAs with Sidak corrections. *, *P* < 0.05; **, *P* < 0.01; ***, *P* < 0.001; ****, *P* < 0.0001; ns, not significant

**Fig. 3:**
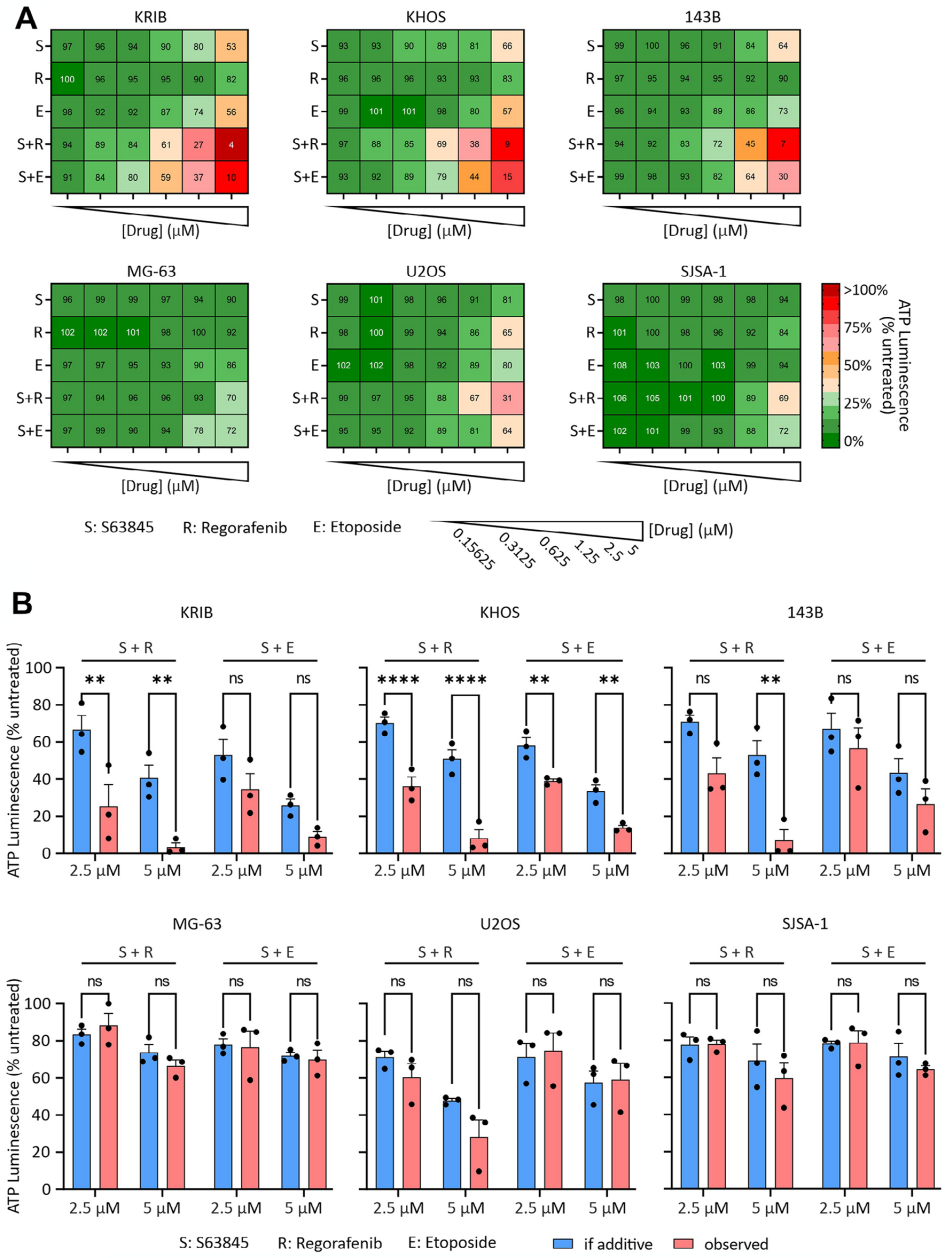
S63845 cooperated with regorafenib or etoposide to reduce osteosarcoma cell viability. Osteosarcoma cells were cultured in growth media containing 0–5 µM of each drug for 48 h. (**A**) Cell viability heatmaps were generated using CellTiter-Glo for cells treated with S63845, regorafenib and etoposide alone or in combination with S63845. Values indicate mean cell viability, normalized to the untreated cells, from three biological replicates. (**B**) Expected additive effects were calculated as fractional products using the viability of cells treated by specified single drugs and were compared to the observed viability of the cells in response to the corresponding drug combinations. Data represents mean +/- SEM from *n* = 3 independent replicates. Statistical analysis of differences between the calculated (assuming additive interactions) and observed viabilities were compared using ANOVAs with Sidak corrections. *, *P* < 0.05; **, *P* < 0.01; ***, *P* < 0.001; ****, *P* < 0.0001; ns, not significant

**Fig. 4:**
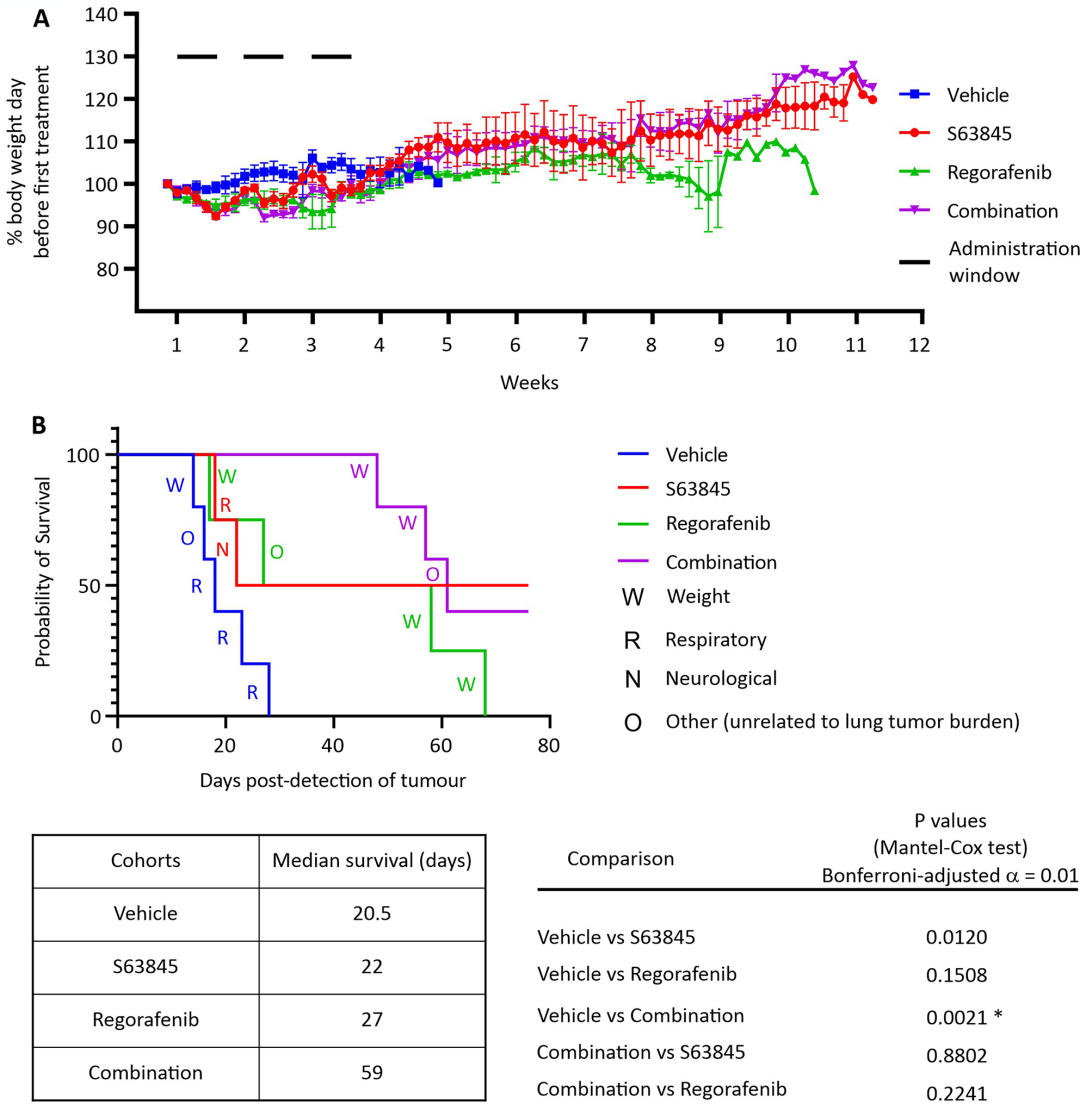
S63845, alone or in combination with regorafenib, improves the survival of mice bearing 143B-luc pulmonary metastases. Luciferase-expressing human 143B osteosarcoma cells were implanted intravenously into nude mice. Specified regimens were commenced when lung metastases became detectable (bioluminescence reached 5 × 10^4^ RLU). (**A**) Mouse body weight was monitored daily thereafter, until euthanasia was required or the pre-determined endpoint of the experiment. *n* = 5 for vehicle and combination, and 4 for S63845 and regorafenib, +/- SEM. (**B**) A Kaplan-Meier survival curve was plotted to illustrate the survival time of each group. The reasons for euthanasia of each mouse were recorded. Log-rank (Mantel-Cox) tests were performed to compare the survival of osteosarcoma-bearing mice between specified groups from the time lung tumors became detectable until euthanasia was required or endpoint of the experiment. After a Bonferroni correction was applied for multiple (five) comparisons, the threshold for statistical significance (α) was 0.01. *, *P* < 0.01

### BH3 mimetics induce caspase-dependent cell death in osteosarcoma cells

CellTiter-Glo 2.0 viability assays measure ATP levels as a surrogate marker for viable, metabolically active cell numbers, which are influenced by cell death and proliferation. Furthermore, this assay does not provide insights into the mode of cell death. Therefore, we aimed to determine whether BH3-mimetic treatment induced caspase-dependent apoptosis in osteosarcoma cells. We assessed cell death using Annexin V/propidium iodide (PI) staining, with and without the presence of the pan-caspase inhibitor Q-VD-OPh (Q-VD), following treatment duration of two and six-hours. This assay distinguishes the early phase of classical apoptosis (cells are Annexin V positive but propidium iodide negative) from late stage apoptosis and lytic forms of cell death (when cells stain positive with both Annexin-V and PI) [48]. The combination of A-1331852 and S63845 were selected to induce cell death, as dual inhibition of BCL-x_L_ and MCL-1 was the most potent combination when measured by CellTiter-Glo (Fig. 1). None of the tested cell lines responded to to 1 µM S63845 and A-1331852 when used as single agents within six hours (Fig. 2). Encouragingly, the combined treatment induced moderate to almost complete cell death in all cell lines, which could be significantly inhibited by Q-VD. The data from the 2-hour treatment demonstrated that the S63845/A-1331852-induced cell death occurred rapidly, as expected for cell death stimulated by BH3-mimetics. These results revealed that while inhibition of either MCL-1 or BCL-x_L_ alone had little effect on viability, dual inhibition induced rapid and robust cell death in osteosarcoma cells which was predominantly caspase-dependent apoptosis.

### S63845 synergizes with second-line osteosarcoma treatments to reduce osteosarcoma cell viability

The above findings suggested a significant role for MCL-1 in the survival of osteosarcoma cells. If BH3-mimetics are to be considered as possible treatments for osteosarcoma patients, they are likely to be combined with currently used therapies. Consequently, we aimed to investigate whether S63845 could cooperate with existing second-line treatments etoposide and regorafenib [7, 8, 49, 50]. Osteosarcoma cells were treated with 0–5 µM of each drug alone or in combination with S63845 at a fixed 1:1 ratio and cell viabilities were measured 48-hours later by CellTiter-Glo. Although the combined treatment of S63845 with either regorafenib or etoposide induced concentration-dependent cytotoxicity, the effects were more pronounced in KRIB, KHOS and 143B cell lines than in MG-63, U2OS and SJSA-1 cells (Fig. 3A). We then compared the observed effects of the combined treatments, at 2.5 µM or 5 µM for each drug, to the calculated effects based on the assumption of additivity (as calculated using the fractional product formula [51]). S63845 cooperated with etoposide only in KHOS cells but cooperated with regorafenib in KRIB, KHOS and 143B cells (Fig. 3B). This observation prompted us to focus on co-treatment with the MCL-1 inhibitor plus regorafenib in subsequent animal studies.

### S63845 plus regorafenib inhibits osteosarcoma growth in vivo

Finally, we determined whether the cooperation we observed between the MCL-1 inhibitor S63845 and regorafenib in vitro could also inhibit osteosarcoma lung metastases growth in vivo. We previously reported an aggressive osteosarcoma metastasis model that involved intravenous injection of 143B cells, leading to the formation of lung metastases in under two weeks [52]. Briefly, mice were intravenously injected with luciferase-expressing 143B osteosarcoma cells (143B-luc) and allocated into treatment groups when tumors were detectable in the lungs. We selected drug doses and frequency of administration that have been published to be well tolerated by nude mice as single agents; 25 mg/kg S63845 twice per week [53] and 30 mg/kg regorafenib three times per week [54]. As expected, all but two mice (which were excluded from the experiment) developed lung metastases within two weeks after tumor implantation. Mice treated with S63845 and regorafenib, either as single agents or in combination, exhibited growth retardation or temporary weight loss in response to the drug administration (Fig. 4A). Notably, the two rest days each week appeared to alleviate therapy-related toxicity, resulting in a slight weight gain, especially in the S63845 and the combination groups (Fig. 4A). This pattern suggested that while the drugs induced an immediate adverse effect on growth and/or weight, there was a partial recovery period when the treatment was paused, reflecting the dynamic response of the mice to the treatment regimen.

Consistent with our previous observations, all of the untreated mice died within four weeks of lung tumor detection (Fig. 4B). Treatment with S63845 plus regorafenib significantly improved survival compared to the vehicle group. Two of the four mice that received regorafenib as a sole agent survived for more than eight weeks but the other two died early during the experiment. Similarly, two mice treated just with S63845 died by 22 days after metastases were detected but the other two survived until the experimental endpoint. Although the combined treatment yielded a median survival time more than double those achieved by administering S63845 or regorafenib treatment alone, statistical analyses of the survival curves could not exclude this difference being due to chance. The prolonged survival of the mice in the combined treatment group was attained without an associated increase in toxicity compared to the single-drug groups, as evidenced by similar body weights fluctuations during treatment and subsequent weight restoration after treatments ceased. These findings highlight the clinical potential of the drug combination, demonstrating its efficacy in extending survival while maintaining a favourable safety profile.

## Discussion

Multiple drug classes have undergone clinical trials to assess their safety and efficacy in treating osteosarcoma, but little progress has been made in improving survival rates for several decades [55]. BH3-mimetics are a drug class that is relatively unexplored as a potential therapy for osteosarcoma compared to treatments such as immunotherapy and tyrosine kinase inhibitors [55]. There have been few pre-clinical studies that have explored the utility of BH3-mimetics to treat osteosarcoma and all were conducted in a small number of cell lines [30–32, 56].

In this study we showed that inhibition of MCL-1 (with S63845) combined with inhibition of BCL-2 (with ABT-199) or BCL-x_L_ (with A-1331852) consistently reduced the viability of osteosarcoma cells in vitro when drugs were applied at low micromolar concentrations (Fig. 1). Osteosarcoma cells were extremely sensitive to dual inhibition of MCL-1 and BCL-x_L_, as evidenced by rapid apoptosis at low concentrations (Fig. 2), whilst combined BCL-x_L_ and BCL-2 antagonism had minimal impact on cell viability. This suggests that osteosarcoma cells predominantly rely on MCL-1 for their survival, while BCL-x_L_ and BCL-2 complement MCL-1 in maintaining osteosarcoma cell survival. In line with our findings, co-inhibition of MCL-1 and BCL-x_L_ showed stronger cytotoxicity in many sarcomas [57] and other cell lines, including those derived from colorectal cancer (CRC) [58] and cervical cancer [45], compared to co-inhibition of BCL-2/BCL-x_L_ or MCL-1/BCL-2. Despite the different BH3-mimetics being used in different studies, the co-dependency on MCL-1 and BCL-x_L_ for cell survival appeared to be common across many other solid cancer types [35, 59]. Current understanding of the relationship between BCL-2 family member expression levels and the sensitivities to BH3-mimetics remains controversial. This could be attributed to the complexity arising from the functional redundancy among anti-apoptotic BCL-2 family proteins, where BCL-2, BCL-x_L_ and MCL-1 can compensate for each other when one or two are deficient [60], possibly by binding to the pro-apoptotic proteins BIM and BAX [37, 40, 43] (Supplementary Fig. 1). For instance, it has been reported that high levels of BCL-2 expression were associated with increased sensitivity to ABT-199 in multiple myeloma (MM), but those cells were resistant when BCL-x_L_ or MCL-1 were also expressed [61]. While some studies have reported that drug sensitivities to BH3-mimetics are related to expression levels of specific BCL-2 family proteins, especially the protein targeted by the drug or other pro-survival relatives [35–37, 39–47], such correlations were either weak or absent in particular cancers, including CRC [62] and AML [63], and B-cell lymphoma [64]. In our study, the most marked difference in BH3 mimetic sensitivity by osteosarcoma cell lines was their sensitivity to MCL-1 antagonism (by S63845). In the osteosarcoma cells we used, S63845 sensitivity correlated with MCL-1 expression, as had been previously noted in non-small cell lung cancer [39]. Presumably, in the less sensitive cell lines, the levels of other pro-survival relatives were sufficient to counteract the impact on BAX/BAK activation of S63845 plus the BH3-only proteins expressed by those cells (Supplementary Fig. 1). Levels of BH3-only proteins, especially PUMA, also correlated with S63845 responsiveness in osteosarcoma cells, reinforcing the importance of the balance of pro-apoptotic versus anti-apoptotic signals in determining cell fate. Apoptosis results from an integration of the influence of multiple pro- and anti-apoptotic BCL-2 relatives on BAX and BAK, which control mitochondrial outer membrane integrity [66]. As previous studies revealed that intact BAX/BAK machinery is required for the anti-tumor effects of BH3-mimetics [65, 66], we confirmed the expression of BAX and BAK in all tested osteosarcoma cell lines. Although knock-out of BAX/BAK caused resistance of some cancer cell lines to the inhibition of the BCL-2 pro-survival proteins [36, 66, 67], researchers revealed that the expression levels of BAX and/or BAK across cell lines from the same origin showed no correlation with the sensitivities to the BH3-mimetics [36], consistent with our observations in the context of osteosarcoma cell lines. Further analyses using cells from more osteosarcomas will be required to determine the feasibility of predicting responsiveness of individual tumors to BH3-mimetics through measuring expression levels of BCL-2 family members like MCL-1 and PUMA. BH3-profiling [68] or in vitro sensitivity testing [69] may also enable predictions of BH3-mimetic responses in these tumors.

Although apoptosis was induced by co-inhibition of MCL-1 and BCL-x_L_, as evidenced by the ability of the caspase inhibitor Q-VD to substantially protect from cell death, our finding that Q-VD failed to completely inhibit MCL-1/A-1331852-induced cell death implies that other forms of cell deaths may be involved. Although the exact mechanism remains unclear, caspase-independent cell death (CICD) could possibly occur as a result of MOMP followed by loss of mitochondria function during the co-inhibition of MCL-1 and BCL-x_L_ [70]. Such BH3-mimetic-induced CICD has been reported in several studies. In diffuse large B-cell lymphoma, researchers attributed the CICD induced by ABT-199 or S63845 to JNK activation [71]. Another study also demonstrated that CICD contributed to a portion of U2OS cell death induced by BH3-mimetics, and was accompanied by MOMP, mtDNA release, and subsequent inflammatory cGAS-STING-dependent interferon response [72]. One study demonstrated that deletion or pharmacologic inhibition of MCL-1-induced DNA damage and cell-cycle arrest regardless of an intact apoptotic pathway [73], suggesting that non-apoptotic functions of the BCL-2 family antagonism also contribute to the overall tumor-suppressing effects of the BH3-mimetics. Mitochondrial dysfunction is a key factor in the intrinsic apoptosis pathway mediated by BCL-2 family proteins. This dysfunction is often associated with elevated levels of reactive-oxygen species (ROS) [74, 75], which may subsequently induce one or more forms of CICD, such as caspase-independent apoptosis, ferroptosis, autophagy, and necroptosis [76].

Although osteosarcoma cells were highly sensitive to combined antagonism of MCL-1 and BCL-x_L_, dual targeting of these proteins can be hepatotoxic [77], which could complicate clinical translation of this finding. We therefore modeled the outcome of combining MCL-1 inhibition with agents currently used to treat osteosarcoma patients. We observed strong in vitro cooperation of S63845 combined with second-line osteosarcoma treatments (Fig. 3A, B). The cooperation between S63845 and etoposide was consistent with numerous studies that have previously reported the cooperation of BH3 mimetics with cytotoxic chemotherapies [78–82]. Only one other study has reported on the interaction of S63845 and regorafenib. Those authors described how MCL-1 inhibition sensitized treatment-resistant colorectal cancer cells to regorafenib [83]. Song et al. found that regorafenib treatment resulted in strong binding of MCL-1 to PUMA and that inhibition of MCL-1 with S63845 freed PUMA to facilitate regorafenib-induce apoptosis in MCL-1 knock-in colorectal cancer cells [83, 84]. Regorafenib has been published to promote FOXO3a-mediated expression of the pro-apoptotic BH3-only protein BIM [85]. It is possible that regorafenib’s ability to sensitize osteosarcoma cells to S63845-mediated death in vitro may reflect its ability to alter the balance between pro- and anti-apoptotic members of the BCL-2 family (Supplementary Fig. 1).

New and better therapies against osteosarcoma metastases are urgently needed to address both innate and acquired drug resistance. Clinical studies have demonstrated the potential of regorafenib, as a single agent, in osteosarcoma patients with metastatic disease after failure of conventional chemotherapy, showing positive impact in delaying disease progression [7, 8]. Numerous clinical trials of MCL-1-targeting drugs are underway or completed but not yet reported, including some evaluating a derivative of S63845 (MIK665, also known as S64315). Unfortunately, other MCL-1 inhibitors, namely ABBV-467 and AMG-397, provoked signs of heart damage in clinical trials, but hopefully a therapeutic window can be defined so that this class of agents can progress to clinical use [86]. Other approaches such as targeting CDK9, which has a significant impact on MCL-1 expression, also hold promise in this context [87].

To test whether the combined treatment using S63845 and regorafenib could reduce the progress of osteosarcoma lung metastases in vivo, we compared four groups of mice receiving vehicle, single or combined treatments. We demonstrated safety of combining S63845 and regorafenib in nude mice bearing osteosarcoma lung metastases. Co-treatment with S63845 and regorafenib nearly tripled the median survival of mice bearing 143B lung tumors. Exploring the mechanism by which S63845 and regorafenib cooperated to kill osteosarcoma cells in vitro and extend survival in vivo was beyond the scope of this study. This mechanism may well be multifaceted, because regorafenib targets numerous kinases [10, 88]. Some of these enzymes provoke signaling pathways within cancer cells that drive their survival and proliferation [85], which could account for the in vitro cooperation that we observed between regorafenib and S63845. Other regorafenib targets, like VEGFR1-3, are expressed on endothelial cells and act to promote angiogenesis, which could drive tumor growth, so their inhibition by regorafenib could have contributed to its cooperation with S63845 that we noted in vivo.

Encouragingly, the combined treatment improved survival without exacerbating drug toxicity compared to the single-drug groups. However, it is important to note that murine MCL-1 has a lower affinity for S63856 than human MCL-1, and mice bearing the human rather than murine MCL-1 gene (“huMCL-1 mice”) had a lower maximal tolerated dose of S63845 than wild type animals [89]. Hence, the mice in our experiment may have tolerated this drug, alone and with regorafenib, better than human patients. A valuable next step would be to evaluate the S63845/regorafenib combination for safety and efficacy in huMCL-1 mice harboring osteosarcoma metastases. If such osteosarcoma models can be developed and tested, they may provide insight into a possible therapeutic window in humans for this co-treatment. With increasing use of regorafenib in osteosarcoma treatment and the observation of regorafenib resistance, as reported in CRC [83], development of combined therapy has become necessary to expand our options for fighting osteosarcoma metastasis. Together with our in vitro finding that MCL-1 plays a predominant role in maintaining osteosarcoma cell survival, as it does in many other types of cancers, our in vivo data highlight the clinical potential of the combining MCL-1 inhibition with regorafenib in the treatment of metastatic osteosarcoma.

## Conclusions

In summary, the present study demonstrated the critical role of MCL-1 in osteosarcoma cell survival and highlighted the potential therapeutic efficacy of targeting MCL-1 in combination with regorafenib. While co-inhibition of BCL-2 and BCL-x_L_ failed to induce osteosarcoma cell death, co-inhibition of MCL-1 with either BCL-2 or BCL-x_L_ led to rapid apoptosis, underscoring the predominant role of MCL-1 in osteosarcoma cell survival. Furthermore, by inhibiting MCL-1, we observed significant cooperation with regorafenib, both in vitro and in vivo, improving survival outcomes in mouse models of osteosarcoma metastasis. These findings suggest that combining MCL-1 inhibitors with regorafenib could represent a promising strategy for treating metastatic osteosarcoma, addressing the limitations of current second-line treatments. Importantly, the combination therapy achieved these effects without increasing toxicity, making it a feasible approach for further clinical investigation. Given the urgent need for new therapeutic options for osteosarcoma, particularly in patients with metastatic disease, our results support the exploration of MCL-1-targeted treatments to improve patient outcomes in this challenging cancer type.

## Electronic supplementary material

Below is the link to the electronic supplementary material.

Supplementary Material 1

## Data Availability

Data is provided within the manuscript or supplementary information files.

## Abbreviations

AML: Acute Myeloid Leukemia
BCL: 2-B-cell lymphoma 2
BCL: x_L_-B-cell lymphoma-extra large
BH3: BCL-2 homology 3
CLL: Chronic Lymphocytic Leukemia
CRC: Colorectal Cancer
CSF: 1R-Colony-Stimulating Factor-1 Receptor
MAP: Methotrexate, Doxorubicin, Cisplatin
MCL: 1-Myeloid Cell Leukemia-1
PDGFRs: Platelet-Derived Growth Factor Receptors
TIE2: Tyrosine Kinase with Immunoglobulin-like and Epidermal Growth Factor-like Domains 2
VEGFRs: Vascular Endothelial Growth Factor Receptors
