# Supplementary material for: Osteosarcoma cells depend on MCL-1 for survival, and osteosarcoma metastases respond to MCL-1 antagonism plus regorafenib in vivo
Source: BMC Cancer. 2024 Nov 4;24:1350. doi: 10.1186/s12885-024-13088-7 (PMC11533409; doi:10.1186/s12885-024-13088-7)
Supplement: Supplementary file 1 — Supplementary Material 1 [file 12885_2024_13088_MOESM1_ESM.docx]

**Osteosarcoma cells depend on MCL-1 for survival and osteosarcoma metastases respond to MCL-1 antagonism plus regorafenib *in vivo***

Yanhao Ji^1^, Michael A. Harris^1,2^, Lucas M. Newton^1,3^, Tiffany J. Harris^4^, W. Douglas Fairlie^1,4,5^, Erinna F. Lee^1,4,5^ & Christine J. Hawkins^1^ (corresponding author)

1. La Trobe Institute for Molecular Science, La Trobe University, Melbourne, VIC, 3086, Australia
2. Peter MacCallum Cancer Centre, Melbourne, VIC, 3000, Australia
3. Swinburne University, Hawthorn, VIC, 3122, Australia
4. Olivia Newton-John Cancer Research Institute, Heidelberg, VIC, 3084, Australia
5. School of Cancer Medicine, La Trobe University, Melbourne, VIC, 3086, Australia


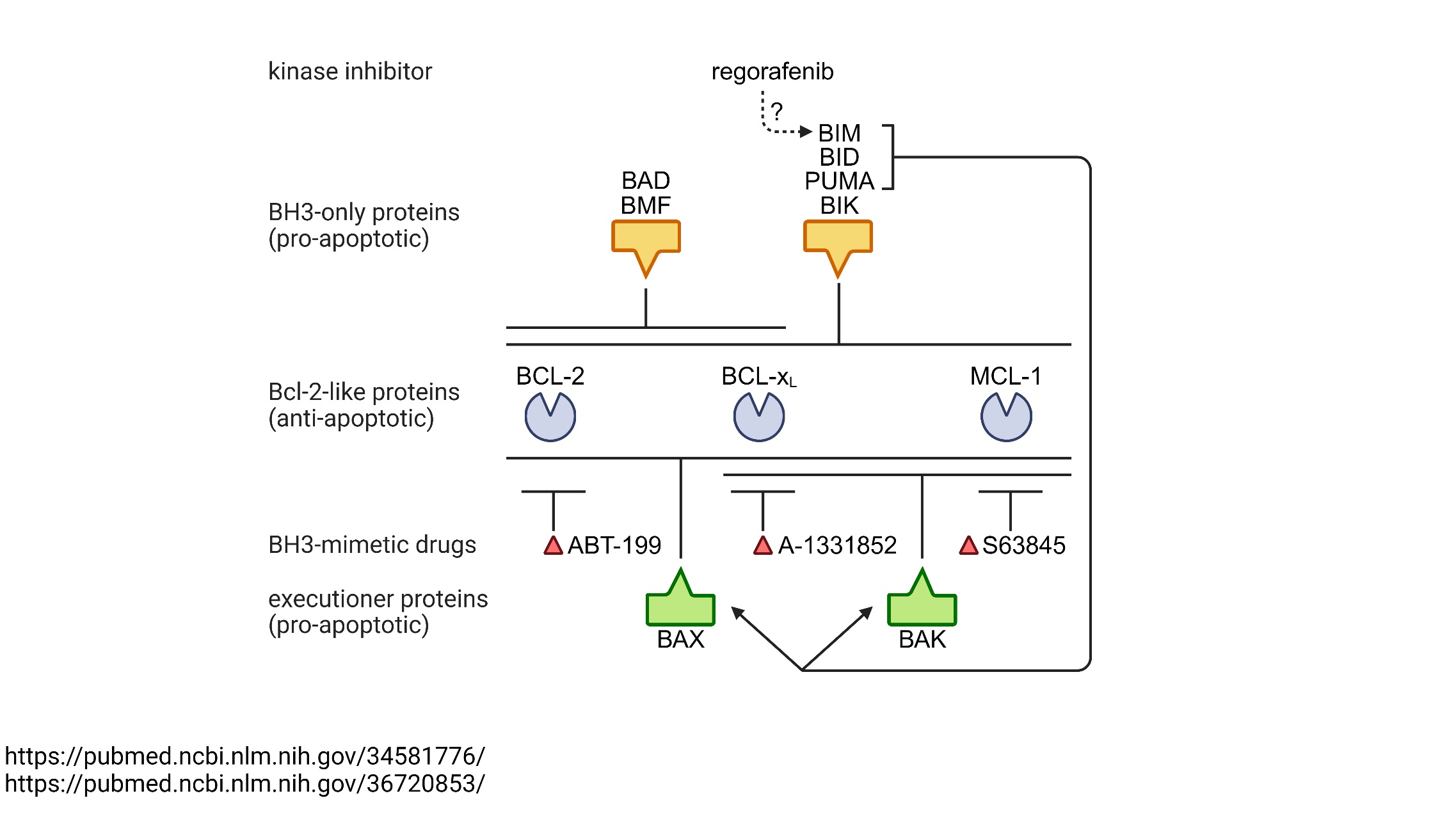


**Supplementary Figure 1**: Model illustrating interactions between proteins and drugs analyzed in this study (Fairlie WD and Lee EF, Biochem Soc Trans 49:2397-2410, 2021). Regorafenib has been shown to boost Bim expression (Sun B et al, Cell Death Discov 9:37, 2023) but may also interact with Bcl-2 family proteins via other mechanisms. Created in BioRender.com


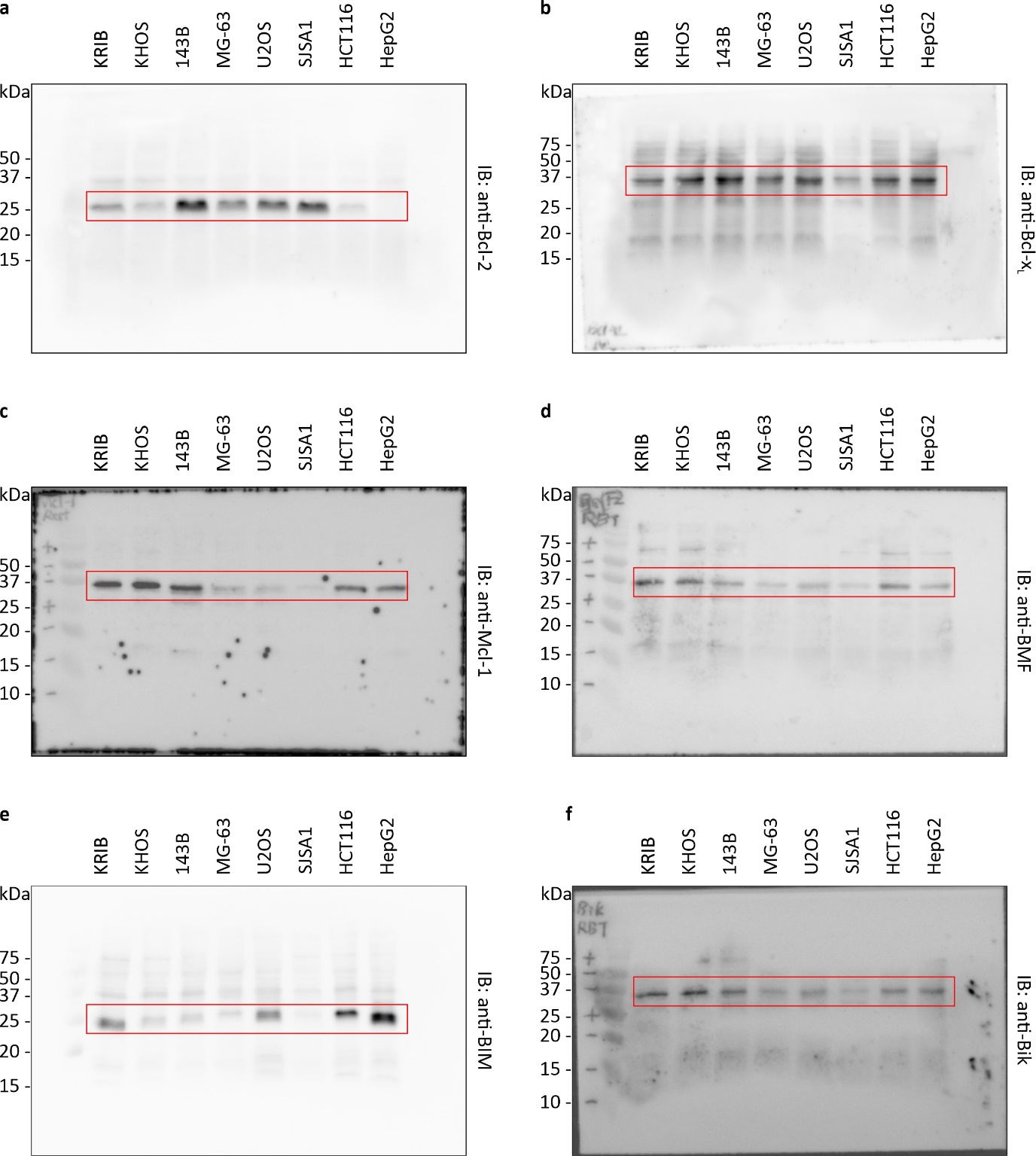


**Supplementary Figure 2:** Uncropped western blots for Figure 1B. Expression of Bcl-2 family proteins were assessed by western blot. Each membrane was immunoblotted by: **a** anti-Bcl-2 antibody; **b** anti Bcl-x_L_ antibody; **c** anti-Mcl-1 antibody; **d** anti-BMF antibody; **e** anti-BIM antibody; and **f** anti-Bik antibody. The red-outlined rectangle highlights the cropped area shown in Figure 1B. The remaining uncropped figures for Figure 1B are provided in Supplementary Figure 2.

**
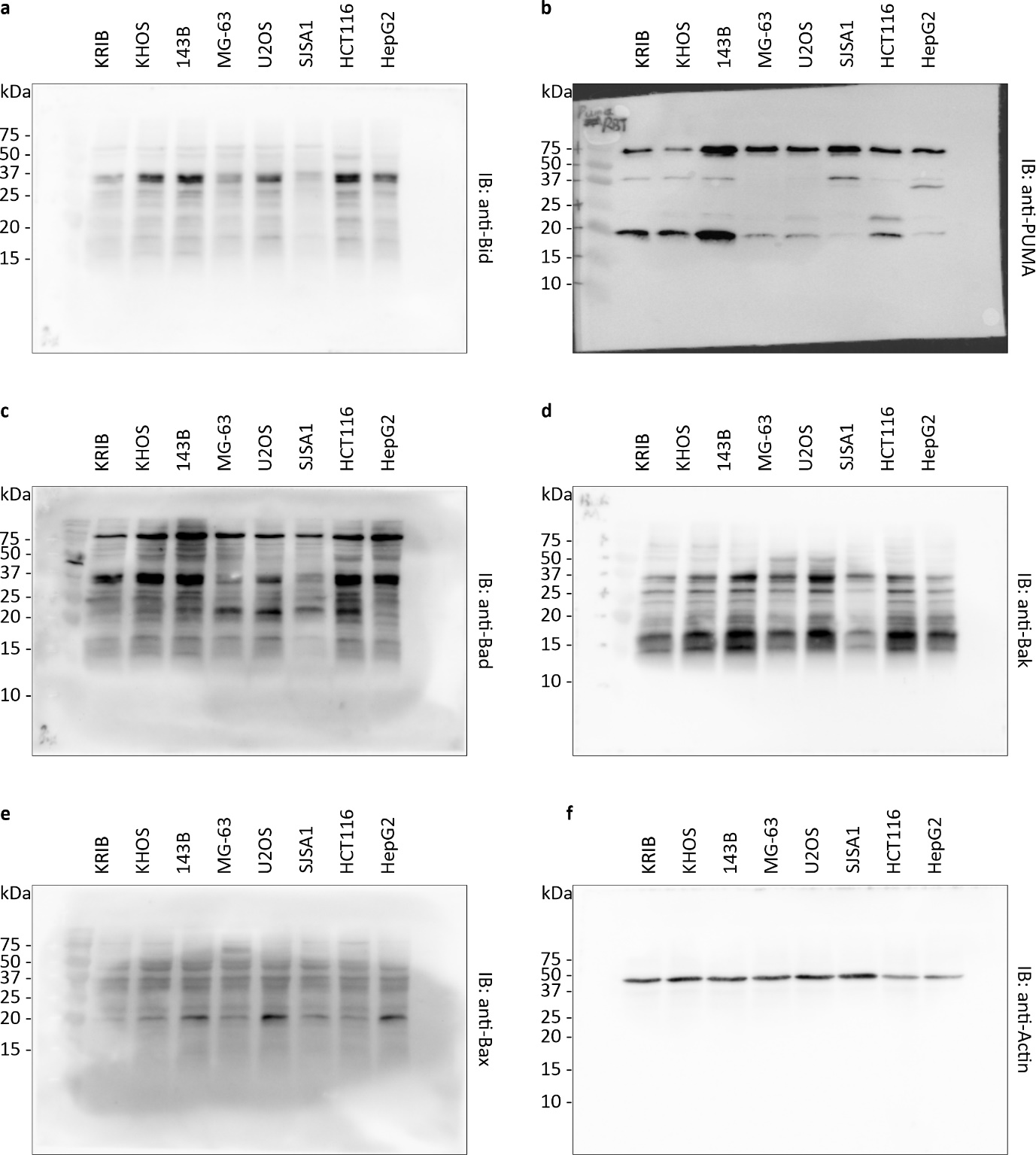
**

**Supplementary Figure 2:** Uncropped western blots for Figure 1B. Expression of Bcl-2 family proteins were assessed by western blot. Each membrane was immunoblotted by: **a** anti-Bid antibody; **b** anti PUMA antibody; **c** anti-Bad antibody; **d** anti-Bak antibody; **e** anti-Bax antibody; and **f** anti-Actin antibody. The red-outlined rectangle highlights the cropped area shown in Figure 1B.
